# Assessment of Value of Neighborhood Socioeconomic Status in Models That Use Electronic Health Record Data to Predict Health Care Use Rates and Mortality

**DOI:** 10.1001/jamanetworkopen.2020.17109

**Published:** 2020-10-22

**Authors:** Alejandro Schuler, Liam O’Súilleabháin, Gina Rinetti-Vargas, Patricia Kipnis, Fernando Barreda, Vincent X Liu, Oleg Sofrygin, Gabriel J. Escobar

**Affiliations:** 1 Systems Research Initiative, Kaiser Permanente Division of Research, Oakland, California; 2 TPMG Consulting Services, Oakland, California; 3 Intensive Care Unit, Kaiser Permanente Medical Center, Santa Clara, California

## Abstract

**Importance:**

Prediction models are widely used in health care as a way of risk stratifying populations for targeted intervention. Most risk stratification has been done using a small number of predictors from insurance claims. However, the utility of diverse nonclinical predictors, such as neighborhood socioeconomic contexts, remains unknown.

**Objective:**

To assess the value of using neighborhood socioeconomic predictors in the context of 1-year risk prediction for mortality and 6 different health care use outcomes in a large integrated care system.

**Design, Setting, and Participants:**

Diagnostic study using data from all adults age 18 years or older who had Kaiser Foundation Health Plan membership and/or use in the Kaiser Permantente Northern California: a multisite, integrated health care delivery system between January 1, 2013, and June 30, 2014. Data were recorded before the index date for each patient to predict their use and mortality in a 1-year post period using a test-train split for model training and evaluation. Analyses were conducted in fall of 2019.

**Main Outcomes and Measures:**

One-year encounter counts (doctor office, virtual, emergency department, elective hospitalizations, and nonelective), total costs, and mortality.

**Results:**

A total of 2 951 588 patients met inclusion criteria (mean [SD] age, 47.2 [17.4] years; 47.8% were female). The mean (SD) Neighborhood Deprivation Index was −0.32 (0.84). The areas under the receiver operator curve ranged from 0.71 for emergency department use (using the LASSO method and electronic health record predictors) to 0.94 for mortality (using the random forest method and electronic health record predictors). Neighborhood socioeconomic status predictors did not meaningfully increase the predictive performance of the models for any outcome.

**Conclusions and Relevance:**

In this study, neighborhood socioeconomic predictors did not improve risk estimates compared with what is obtainable using standard claims data regardless of model used.

## Key Points

**Question:**

Is neighborhood socioeconomic status useful for predicting 1-year health care use rates and mortality in the general adult population?

**Findings:**

In this diagnostic study of data from 2 951 588 patients, neighborhood socioeconomic status did not improve the predictive performance of models regardless of modeling strategy.

**Meaning:**

Neighborhood socioeconomic status may not improve prediction models for short-term use and mortality in insured populations.

## Teaser

This diagnostic study assesses the value of including neighborhood socioeconomic status in models that use electronic health record (EHR) data to predict health care use rates and mortality among adults in a large integrated care system.

## Introduction

Prediction models are widely used in health care and health services research.^1,2,3,4,5,6^ Models can be used for individual risk prediction, risk adjustment, or to aid forecasting for entire populations. Assessing predicted risk is one approach to find patient subgroups that may be targetable by available interventions or who require new interventions to be designed for them.^7,8^ If an existing intervention is equally efficacious among patients in a population, targeting it to those patients most at risk for the outcome prevents the most outcomes for a given cost.^9^

Administrative or claims data, which are widely available, are commonly used for prediction models, particularly outside the research setting.^10^ Claims data include demographic information, diagnosis codes, and date stamps. Although multiple studies have demonstrated improved prediction when other data types are included,^5,11,12,13,14,15,16,17^ administrative data remain most commonly used, perhaps because mandates for the use of other data are rare.

As the proportion of health care encounters taking place in settings with electronic health records (EHRs) has increased, interest in using clinical data (eg, laboratory test results; vital signs; body mass index [BMI], and granular health services information) has also increased. However, clinical data do not tell a complete story about a patient’s health status or future risk and so an increasing number of studies have explored the use of socioeconomic, environmental, and behavioral indicators that are not usually captured explicitly in the EHR but are associated with patient outcomes.^18,19,20^ These social determinants of health may be useful as predictors but are difficult to define, collect, and track, especially at the individual level and at scale.^18,21,22,23,24^

Neighborhood-level data linked to patient records by zip code may also have utility, either as proxies for individual socioeconomic factors or as independent predictors in their own right, but gathering those data and linking them requires substantial effort.^25,26,27^ The key operational question is whether it is worthwhile for health system administrators to mandate collection of these variables for the purposes of risk stratification or risk adjustment.^28^

Neighborhood socioeconomic status (nSES) variables have been reported to improve predictions in some cases but not others. For instance, Molshatzki et al^29^ found that nSES variables improved the prediction of long-term mortality after myocardial infarction but Bhavsar et al^30^ found that an nSES index did not improve prediction of a variety of health care use measures within a 3-year window. Another study suggests that neighborhood-level indicators are not predictive above and beyond individual-level indicators.^18^ Whether these predictors are useful for risk stratification, and, if so, in what contexts remains unclear.

The nSES variables may have different utility depending on the predictive task and the population. We assess the addition of diverse nSES predictors to various risk models to predict 1-year health care use, hospitalization, and mortality in a large, heterogeneous population. However, while our prediction tasks and cohort are diverse, they are not exhaustive. We limit ourselves to 1-year prediction windows, but it is possible that nSES predictors may be more useful in longer term.

We included 3 types of predictors (claims, clinical, and nSES) in prediction models that use 3 complementary machine learning modeling approaches (penalized regression, random forests, and neural networks). The setting for our investigation is Kaiser Permanente Northern California (KPNC), an integrated health care delivery system with comprehensive information systems.

## Methods

This data-only project was approved by the KPNC Institutional Review Board for the Protection of Human Subjects, which waived the individual informed consent requirements.

Under a mutual exclusivity agreement, 9500 salaried physicians of The Permanente Medical Group, Inc., care for 4.3 million Kaiser Foundation Health Plan, Inc. (KFHP) members. Care for these members, which includes all subspecialty care, occurs at 21 hospitals owned by Kaiser Foundation Hospitals, Inc. as well as over 250 medical office buildings. A common medical record number system is used for all patient clinical encounters and administrative transactions, including those involving KFHP members who get care outside the system and nonmembers cared for at KFH emergency departments and hospitals. The comprehensive nature of these information systems, and the ability to extract and use information from them, including diagnostic, laboratory, and health services data, has been documented in multiple publications by KPNC researchers.^13,31,32,33,34^

We scanned KPNC databases and identified all adults aged 18 years or older who had KFHP membership and/or use rates between January 1, 2013 and June 30, 2014 to define the cohort. A set of covariates was defined on the 12-month preperiod interval between July 1, 2013, and June 30, 2014, while the outcomes were defined on the 12-month postperiod interval between July 1, 2014, and June 30, 2015. Each patient’s address was linked to their US census tract which permitted us to then link to the specific administrative, clinical, and nSES variables described below. We removed data from patients with duplicate medical record numbers, death in the preperiod or before, negative cost data, lack of KPNC membership, or missing nSES, diagnosis codes, or age data. Analyses were conducted in fall of 2019.

The 3 categories of independent variables for our prediction models, which are also detailed in the eAppendix in the Supplement, are detailed in Table 1. The first set of predictors (administrative) includes demographic characteristics, categorical aggregated diagnosis codes (related clinical conditions [RCCs]), and a derived real-valued score predictive of future cost (diagnosis cost group). Both RCCs and diagnosis cost group scores are routinely assigned to all patients with a KPNC medical record number on a monthly basis. With respect to RCCs, any accrual of the relevant diagnosis during the last year of the preperiod month sets the value of that RCC to 1, otherwise it is set to 0. Diagnosis cost group scores are likewise assigned monthly based on data from the preceding 12 months.

**Table 1.** Variables Included in Each of the Predictor Categories

Variable^a^	Total number of variables
Administrative	123
Age	1
Sex	1
Diagnosis cost group (predicted cost score)	4
RCCs	117
EHR	18
HbA_1c_	4
BMI	5
abLAPS score	4
COPS2	4
Online patient portal registration status (yes/no)	1
nSES	27
NDI	1
Transportation	6
Housing	2
Job availability	2
Food access	2
Crime	2
Environmental factors	11
Walkability	1

Abbreviations: abLAPS, laboratory-based acute physiology score; BMI, body mass index; COPS2, Comorbidity Point Score, version 2; EHR, electronic health record; NDI, Neighborhood Deprivation Index; nSES, neighborhood socioeconomic status; RCCs, related clinical conditions.

^a^ The provenance, meaning, and summary statistics for each of the nSES variables are described in the eAppendix in the Supplement.

The second set of predictors (EHR) includes a comorbidity score, Comorbidity Point Score, version 2^33^ that is assigned on a monthly basis to all KPNC adult patients and in real time on hospital admission.^35^ This score is calculated based on administrative data, but is integrated with KPNC’s EHR for real-time use. In addition, we include a composite laboratory index, the abbreviated LAPS (Laboratory-based Acute Physiology Score, abLAPS), which is based on the lowest (indicating maximal physiologic derangement) value for 14 laboratory test results over the preceding month; this score is an outpatient modification of a previously reported hospital score^13^ and is assigned to all KPNC adults each month. We also included BMI and hemoglobin A1c (HbA_1c_). Last, we included an indicator of whether a patient had registered with the KPNC online patient portal.

The third set of predictors (nSES) includes 27 indicators of neighborhood socioeconomic status relating to transportation, housing, jobs, food access, crime, environment, and walkability. These variables were obtained from the US Department of Agriculture, US Environmental Protection Agency, California Environmental Protection Agency and the National Oceanic and Atmospheric Administration (eTable 4 in the Supplement).

Some clinical scores and lab values could be observed at multiple points for each patient in the preperiod. We aggregated these scores to the mean at monthly points. The mean monthly values were then aggregated to the mean overall, the last observed, the maximum observed and the trend of a linear regression over the 12-month preperiod. Another variable was created for BMI that accounted for the difference between the maximum and the mean. When missing throughout the entire record, BMI was imputed to 20 and HbA_1c_ was imputed to 5 mmol/L.

We defined 7 dependent variables for the 1-year postperiod for our analyses: 5 encounter counts (doctor office visits, virtual visits,^36^ emergency department [ED] visits, elective hospitalizations [those that did not begin in the ED], and nonelective hospitalizations [those that began in the ED]), cost, and mortality. Mortality was ascertained from KPNC databases, lists of decedents provided by the Social Security Administration, and State of California Death Certificate data. For the remaining outcomes, we used KPNC databases, with cost being the sum of KFHP expenditures for a patient for prescriptions and care received in the clinic, ED, operating room, emergency department, hospital, ancillary settings (radiology or laboratory), and in outpatient settings. To make all analyses consistent with the binary analysis for mortality, encounter counts and cost were binarized at the 80th percentile of their distributions for the main analysis. This cutoff was chosen by clinical collaborators based on heuristic calculations of how many patients would be flagged as at risk each year at a given threshold and how many patients the health system would have the capacity to include.

For model development, we randomly split the aggregated patient-level data set into 50/50 training and test samples. We used machine learning neural networks, random forests, and least absolute shrinkage and selection operator penalized regression to develop prediction models based on the training data for each of the 7 outcomes described. Details on each of these approaches are available in eTable 5 and the eAppendix in the Supplement.

### Statistical Analysis

For each outcome, we assessed the performance of models using (1) only the administrative predictors, (2) the administrative plus the nSES predictors, (3) the administrative plus the EHR predictors, and (4) using all predictors. Model performance was evaluated using area under the receiver operator curve, area under the precision-recall curve, Brier Score, and McFadden pseudo-*R*^2^, all as calculated in the held-out test set. McFadden pseudo-*R*^2^ was calculated using the likelihood of the test data under a binomial distribution (conditioned on the observed predictors) given the estimated probabilities from the model.

Our analysis follows the Transparent Reporting of a Multivariable Prediction Model for Individual Prognosis or Diagnosis (TRIPOD) reporting guideline^37^ except in reporting unadjusted analyses (not relevant to our aims) and in presenting the full prediction models (eg, coefficients, weights). Our models are meant to assess the utility of nSES predictors and are not validated for use in practice and we fit many complex models, which are not practical to fully describe in natural language.

## Results

Data from a total of 2 951 588 patients were included in this study. 47.8% of patients were female; 52.2% were male. The mean (SD) Neighborhood Deprivation Index was −0.32 (0.84) and mean (SD) age was 47.2 (17.4) years. Additional summary statistics for the test cohort are in Table 2 and further details are available in eTables 1-3 of the Supplement.

**Table 2.** Summary Statistics for the Test Cohort

Variable	Mean (SD)	
	Preperiod	Postperiod
Population, total No.	1 475 559	1 465 343
Age, y	47.20 (17.39)	47.00 (17.24)
Male sex, %	48	48
Charlson score	0.49 (1.24)	0.47 (1.19)
COPS2	13.16 (12.94)	12.88 (12.08)
abLAPS	1.94 (4.50)	1.88 (4.34)
Diabetes (RCC 7), %	7.5	8.1
HbA_1c_	4.98 (0.62)	4.98 (0.61)
CHF (RCC 59), %	1.5	1.7
COPD (RCC 77), %	7.8	8.9
Invasive cancer (RCCs 2,3,4), %	1.7	1.8
CKD (RCC 87), %	4.5	4.9
Major infection (sepsis, community-acquired pneumonia, RCC 1), %	8.2	8.7
BMI	25.43 (6.40)	25.43 (6.40)
NDI	−0.16 (0.84)	−0.16 (0.84)
In-person outpatient visits	3.59 (5.89)	3.50 (5.89)
Virtual visits	3.30 (6.89)	3.25 (6.86)
ED visits	0.24 (0.83)	0.24 (0.87)
ED visits (≥4), %	0.93	1
Elective hospitalization	0.03 (0.21)	0.03 (0.21)
Elective hospitalization (≥2), %	0.32	0.32
Elective hospitalization (ever), %	2.7	2.9
Nonelective hospitalization	0.04 (0.26)	0.04 (0.28)
Nonelective hospitalization (≥2), %	0.56	0.66
Nonelective hospitalization (ever), %	2.7	2.9
Cost	4054.88 (14449.06)	4254.68 (16878.31)
≥$15 000, %	5.9	6
≥$100 000, %	0.28	0.35

Abbreviations: abLAPS, laboratory-based acute physiology score; BMI, body mass index (calculated as weight in kilograms divided by height in meters squared); CHF, congestive heart failure; CKD, chronic kidney disease; COPD, chronic obstructive pulmonary disease; COPS2, Comorbidity Point Score, version 2; ED, emergency department; HbA_1c_, hemoglobin A_1c_; NDI, Neighborhood Deprivation Index; RCC, related clinical conditions.

SI conversion factor: To convert HbA_1C_ to proportion of total hemoglobin, multiply by 0.01.

The performance characteristics of our models are shown in the Figure. The areas under the receiver operator curve ranged from 0.71 for emergency department use (using the LASSO method and electronic health record predictors [area under the precision-recall curve, 0.36; Brier Score, .011; and McFadden pseudo-*R*^2^, 0.11]) to 0.94 for mortality (using the random forest method and electronic health record predictors [area under the precision-recall curve, 0.23; Brier Score, .006; and McFadden pseudo-*R*^2^, 0.37]). The exact values are provided in eTable 6 in the Supplement. Table 3 shows the exact area under the receiver operator curve values for the neural network model, which had the best performance in all cases. Calibration plots for all models are shown in the eFigure in the Supplement.

**Figure.**
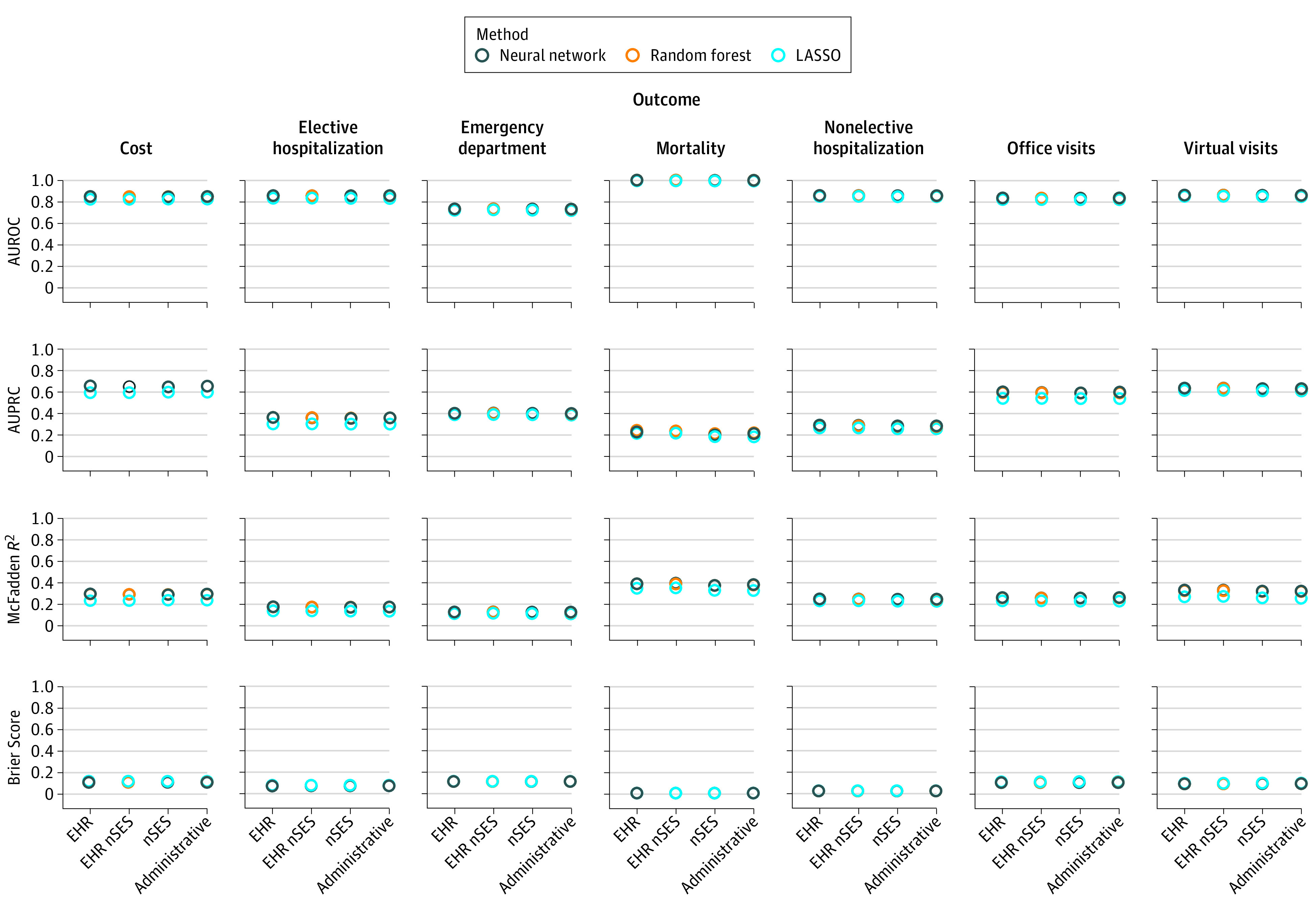
Performance Measures of All Models Across All Predictors Sets and Outcomes Higher is better for all measures except Brier score. AUPRC indicates area under the precision-recall curve; AUROC, area under the receiver operator curve; EHR, electronic health record; LASSO, least absolute shrinkage and selection operator; nSES, neighborhood socioeconomic status.

**Table 3.** Area Under the Receiver Operator Curve of the Best Model (Neural Network) Across All Outcomes and Predictor Sets^a^

Model	Cost	Mortality	Office visits	Emergency department	Nonelective hospital	Elective hospital	Virtual visits
Administrative	0.85	0.94	0.83	0.73	0.85	0.79	0.86
+nSES	0.84	0.94	0.83	0.73	0.85	0.79	0.86
+HER	0.85	0.94	0.83	0.73	0.86	0.79	0.86
+EHR +nSES	0.84	0.94	0.83	0.73	0.86	0.79	0.86

Abbreviations: +nSES, plus neighborhood socioeconomic status; +EHR, plus electronic health records; +EHR +nSES, plus both EHR and nSES.

^a^ Cross-validation standard errors for all figures in this table are below 0.001 due to the size of the data. A full table of performance measures for all methods, predictor sets, and outcomes is available in the eAppendix in the Supplement.

The nSES predictors did not improve the models, regardless of which machine learning method was used. Our EHR predictors also do not meaningfully improve the models. Our results are consistent across each of our performance measures (area under the receiver operator curve, area under the precision-recall curve, McFadden pseudo-*R*^2^, and Brier Score). Although models perform quite differently for different outcomes (because different outcomes may be more difficult or easier to predict), the performance differences between predictor sets and methods are largely consistent within each outcome.

## Discussion

We have developed 7 population-based prediction models using a large contemporary cohort. In addition to using administrative data, we also evaluated the inclusion of nSES variables. We found that adding nSES variables did not result in improved prediction. Although not of primary interest in this study, we also found that our set of EHR predictors did not meaningfully improve the models.

Most studies of nSES only show that these variables are statistically associated with outcomes, not that they improve the ability to risk stratify patients. Predictors that show strong statistical associations with an outcome are not guaranteed to improve the prediction of that outcome relative to some baseline model.^29^

Methods for constructing nSES variables vary greatly across the literature. Some authors collapse many indicators into a single variable, while others examine the effect of including individual predictors like average neighborhood income, education, and occupation. This inconsistency makes it difficult to compare between studies. A review concluded that nSES variables should be examined at their most granular level to improve generalizability.

Because the utility of nSES variables will vary depending on the population, we chose to take a global perspective and include a broad set of patients in our study. If nSES variables were useful for any subsets of this population, we would also expect to see a small improvement in performance at the macro level of our analysis, thus obviating the need for different studies in each population. Since we did not observe this effect, we believe that if there is a subpopulation that benefits from including nSES variables in the prediction of 1-year use rates and mortality, then it may be too small to detect.

The predictive utility of a variable naturally depends on the outcome that is being predicted. For this reason, and to expand on the existing literature, we included a variety of operationally important outcomes (use rates and mortality) in our study.^2,5,38,39,40^ We believe that nSES variables may be more useful for risk stratification in the context of long-term prediction.

The predictive utility of a variable also depends on how effectively the model can make use of it. For instance, if a predictor has a U-shaped association with the outcome, it may not improve risk prediction when using a linear model. Although some previous studies of nSES have used nonparametric models, they have not compared the utility of nSES socioeconomic predictors across a variety of methods. We thus used 3 machine learning methods with state-of-the-art performance to characterize the potential utility of nSES predictors.^3,40^ Although no method was able to extract improvement from the nSES variables, the overall better performance of our neural network suggests the utility of deep learning for clinical prediction modeling.

Our use of a rich set of baseline predictors also distinguishes this study from prior work. However, the predictors are certainly not exhaustive, and in many cases the addition of the EHR variables did not improve performance over the administrative baseline. The performance of our models is comparable with what has been reported in the literature, but performance could likely be improved with the addition of granular labs, prescriptions, or text data. In particular, patient race is an important predictor that we did not use because of heavy missingness in our data. However, including these other predictors would most likely further diminish the utility of nSES predictors. Similarly, we used a basic imputation strategy to fill in missing predictors. Alternative strategies may have resulted in slightly better model performance overall, but the choice of imputation strategy, predictor set, or model in the predictive context does not contribute the kind of biases that would certainly be of concern when estimating associations or causal effects.

### Strengths and Limitations

Study strengths include granular nSES variables, large diverse cohort, and variety of operationally important outcomes. This study also has limitations, which include a scope confined to utility of nSES variables, the 1-year prediction window, and results not being generalizable to other populations. Other studies have found stronger associations between outcomes and individual-level SES than between outcomes and nSES. Using individual SES, however, would require the collection of these variables by the health care system, whereas nSES predictors are available in public data sources.

## Conclusions

Our results show that nSES predictors do not contribute to the predictive power of risk models for 1-year mortality and use outcomes in any of 3 strong modeling approaches, even using a granular set of nSES variables and a larger sample size than ever before attempted. These findings suggest that even if it is possible, it may not be worthwhile to collect and store nSES variables alongside EHR data to assist in risk prediction.

## Notes

Supplement. **eTable 1.** Predictors Obtained From KPNC Databases **eTable 2.** Distribution of RCCs in Validation Cohort **eTable 3.** Neighborhood-Level Predictors and Distribution in Validation Cohort **eTable 4.** URLS of Data Sources **eFigure.** Model Calibration Plots **eTable 5.** Model Outcomes **eTable 6.** Performance Metrics for All Models **eAppendix.** Models
