# Supplementary material for: Assessment of Value of Neighborhood Socioeconomic Status in Models That Use Electronic Health Record Data to Predict Health Care Use Rates and Mortality
Source: JAMA Netw Open. 2020 Oct 22;3(10):e2017109. doi: 10.1001/jamanetworkopen.2020.17109 (PMC7582126; doi:10.1001/jamanetworkopen.2020.17109)
Supplement: Supplement. — eTable 1. Predictors Obtained From KPNC Databases eTable 2. Distribution of RCCs in Validation Cohort eTable 3. Neighborhood-Level Predictors and Distribution in Validation Cohort eTable 4. URLS of Data Sources eFigure. Model Calibration Plots eTable 5. Model Outcomes eTable 6. Performance Metrics for All Models eAppendix. Models [file jamanetwopen-e2017109-s001.pdf]

Supplemental Online Content

Schuler A, O’Súilleabháin L, Rinetti-Vargas G, et al. Assessment of value of neighborhood socioeconomic status in models that use electronic health record data to predict health care use rates and mortality. *JAMA Netw Open*. 2020;3(10):e2017109. doi:10.1001/jamanetworkopen.2020.17109

eTable 1. Predictors Obtained From KPNC Databases
eTable 2. Distribution of RCCs in Validation Cohort
eTable 3. Neighborhood-Level Predictors and Distribution in Validation Cohort
eTable 4. URLS of Data Sources
eFigure 1. Model Calibration Plots
eTable 5. Model Outcomes
eTable 6. Performance Metrics for All Models
eAppendix. Models

This supplemental material has been provided by the authors to give readers additional information about their work.

© 2020 Schuler A et al. *JAMA Network Open*.

eTABLE 1. PREDICTORS OBTAINED FROM KPNC DATABASES	
**VARIABLE**	**DESCRIPTION**
Age	Age, ≥18 years, in pre-period.
Membership	Membership is based on Membership status - a binary response for KP membership or utilization during a given month. Positive membership status in two months separated by 2 months or less with negative membership status is defined as contiguous membership. Membership is contiguous at the extremes if membership status was positive within 3 months of the beginning or end of the period.
Sex	Gender, in pre-period.
Related Clinical Categories (RCCs)	RCC1 – RCC117 (see Table 2A-3 for a description of each RCC).
DxCG	Commercially available index used to predict cost. Assigned monthly to all patients with a KPNC medical record number.
COPS2	COmorbidity Point Score, version 2. Assigned monthly to all patients with a KPNC medical record number based on all accrued diagnoses within the preceding 12 months. In KPNC, this score is also assigned to all patients, in real time. Diagnoses are grouped into Hierarchical Condition Categories (HCCs) and then selected HCCs are incorporated into a regression model, as described in Escobar et al.
abLAPS	Abbreviated Laboratory-based Acute Physiology score. This is a simplified version of the original LAPS score. Assigned monthly to all patients with a KPNC medical record number based on 14 laboratory tests (see Table 1A-3). For each test, points are assigned based on the worst, or most deranged, laboratory test result during the preceding month.
Body Mass Index (BMI)[a]	A person's weight in kilograms divided by the square of height in meters. Values captured include overall mean, last observed, maximum observed, trend over the 12-month pre-period, and the difference between maximum and the mean.
Hemoglobin A1c[b]	Mean, last observed, maximum observed, and trend over the 12-month pre-period.
KP.org registration status	Active registration in Kaiser Permanente's online patient portal (Yes/No), in pre-period.

Footnotes

a Missing values imputed to 20 kg/m2
b Missing values are imputed to 5 mmol/L

© 2020 Schuler A et al. *JAMA Network Open*.

eTABLE 2. DISTRIBUTION OF RCCS IN VALIDATION COHORT		
**RCC**	**DESCRIPTION**	**% IN VALIDATION COHORT WITH THIS RCC**
RCC1	Infections	8.25%
RCC2	Solid Tumors	1.40%
RCC3	Blood and Lymph Neoplasm	0.24%
RCC4	Carcinoma In Situ	0.11%
RCC5	Benign/Uncertain Neoplasm	4.66%
RCC6	Other Neoplasms	3.03%
RCC7	Diabetes Co-Morbidity Level	7.51%
RCC8	Type I Diabetes	1.12%
RCC9	Malnutrition	0.50%
RCC10	Hyperlipidemia and Lipidoses	20.14%
RCC11	Endocrine Conditions	7.80%
RCC12	Excess Weight	9.50%
RCC13	Other Nutritional and Metabolic Conditions	2.70%
RCC14	Liver Intervention and Complications	0.02%
RCC15	Liver Failure	0.10%
RCC16	Biliary and Gallbladder Conditions	0.43%
RCC17	Hepatitis	1.55%
RCC18	Alcoholic Liver, Cirrhosis, and Infarct	0.12%
RCC19	Gastrointestinal Intervention and Complications	4.47E-05
RCC20	Peptic Ulcer and Related Conditions	0.39%
RCC21	Other Gastrointestinal Conditions	18.18%
RCC22	Pancreatic Disorders	0.43%
RCC23	Inflammatory Bowel Disease	0.42%
RCC24	Knee Disorders and Injuries	5.24%
RCC25	Hip Disorders and Injuries	2.62%
RCC26	Back Disorders and Injuries	14.15%
RCC27	Other Musculoskeletal Conditions	10.92%
RCC28	Musculoskeletal Infection	0.17%
RCC29	Inflammatory Musculoskeletal Conditions	1.27%
RCC30	Lower Leg & Foot Disorders and Injuries	7.81%
RCC31	Shoulder & Upper Arm Disorders and Injuries	4.87%
RCC32	Forearm & Hand Disorders and Injuries	5.24%
RCC33	Hemorrhagic Conditions	0.12%
RCC34	Anemia	4.29%
RCC35	Disorders of Immunity	0.21%
RCC36	Cognitive Disorders	1.45%
RCC37	Drug Abuse	0.99%
RCC38	Alcohol Abuse	1.12%
RCC39	Tobacco Use	3.61%
RCC40	Personality Disorders	0.20%
RCC41	Other Mental Conditions	4.63%
RCC42	Psychoses	0.40%
RCC43	Eating Disorders	0.12%
RCC44	Mood and Anxiety Disorders	8.15%
RCC45	Suicide Attempts	0.05%
RCC46	Chromosomal and Developmental Disorders	0.95%
RCC47	Severe Developmental Disability	2.17E-05
RCC48	Neurological Trauma	0.75%
RCC49	Paralysis and Coma	0.12%
RCC50	Seizure Disorders	0.68%

© 2020 Schuler A et al. *JAMA Network Open*.

eTABLE 2. DISTRIBUTION OF RCCS IN VALIDATION COHORT		
**RCC**	**DESCRIPTION**	**% IN VALIDATION COHORT WITH THIS RCC**
RCC51	Degenerative Neurological Conditions	0.42%
RCC52	Myoneural Conditions	0.01%
RCC53	Other Neurological Conditions	4.94%
RCC54	Headache	5.69%
RCC55	Respiratory Arrest	0.65%
RCC56	Cardiac Arrest	0.03%
RCC57	Cardiovascular Intervention and Complications	0.22%
RCC58	Coronary Artery Disease	3.01%
RCC59	Congestive Heart Failure	1.55%
RCC60	Heart Valve and Pericardial Conditions	0.82%
RCC61	Congenital Heart Conditions	0.09%
RCC62	Cardiac Arrhythmias	3.16%
RCC63	Other Heart Conditions	0.17%
RCC64	Hypertension	12.04%
RCC65	Stroke	0.75%
RCC66	Post-Stroke Paralysis	0.47%
RCC67	Sequelae of Cerebrovascular Events	0.26%
RCC68	Cerebro-Vascular Impairment	0.19%
RCC69	Peripheral Atherosclerosis	0.36%
RCC70	Other Peripheral-Vascular Conditions	5.55%
RCC71	Thrombosis/Phlebitis	1.29%
RCC72	Lung Intervention and Complications	2.78E-05
RCC73	Lung Infection	1.04%
RCC74	Lung Congestion and Effusion	0.26%
RCC75	Lung Fibrosis	0.39%
RCC76	Other Lung Conditions	1.87%
RCC77	COPD and Asthma	7.86%
RCC78	Diabetic/Other Retinopathy	1.60%
RCC79	Blindness	0.05%
RCC80	Eye Infection and Inflammation	0.09%
RCC81	Eye Intervention and Complications	2.75%
RCC82	Other Eye Conditions	17.74%
RCC83	Significant ENT Disorders	0.26%
RCC84	Hearing Impairment	2.99%
RCC85	Other ENT Disorders	18.83%
RCC86	Urinary System Intervention and Complications	0.24%
RCC87	Chronic Kidney Disease and Failure	4.51%
RCC88	Bladder and Other Urinary Conditions	3.78%
RCC89	Nephritis	0.25%
RCC90	Urinary System Infection	2.80%
RCC91	Female Genital Conditions	10.15%
RCC92	Male Genital Conditions	4.39%
RCC93	Completed/Terminated Pregnancy	1.91%
RCC94	Other Pregnancy	0.03%
RCC95	Uncompleted Pregnancy	1.00%
RCC96	Severe Burns	6.30E-05
RCC97	Skin Ulcers	0.48%
RCC98	Other Skin Conditions	21.23%
RCC99	Head Injury	0.69%
RCC100	Traumatic Amputation	5.83E-05

© 2020 Schuler A et al. *JAMA Network Open*.

eTABLE 2. DISTRIBUTION OF RCCS IN VALIDATION COHORT		
**RCC**	**DESCRIPTION**	**% IN VALIDATION COHORT WITH THIS RCC**
RCC101	Other Injuries	5.06%
RCC102	Poisoning	1.33%
RCC103	Symptoms	37.59%
RCC104	Short Gestation and Low Birthweight	0.00%
RCC105	Serious Perinatal Conditions	0.00%
RCC106	Single/Multiple Birth	0.00%
RCC107	Other Perinatal Conditions	0.00%
RCC108	Bone Marrow Transplant and Complications	0.02%
RCC109	Artificial Openings	0.18%
RCC110	Amputation Status	0.16%
RCC111	Other V-Codes	0.13%
RCC112	Other Transplant Status and Complications	0.15%
RCC113	Chemical and Radiation Oncology	0.13%
RCC114	Other Screening and History	58.71%
RCC115	Post-Procedural Conditions	12.43%
RCC116	Implant and Device Complications	0.34%
RCC117	Other Complications	1.48%

© 2020 Schuler A et al. *JAMA Network Open*.

eTABLE 3. NEIGHBORHOOD-LEVEL PREDICTORS AND DISTRIBUTION IN VALIDATION COHORT			
**VARIABLE**	**DESCRIPTION**	**PROVENANCE**	**DISTRIBUTION IN VALIDATION COHORT (MEDIAN, MEAN +/- SD)**
NDI	Neighborhood Deprivation Index; First component of Principal Component Analysis of 8 variables: % adult population with less than a high school diploma, % of households earning less than $30,000/annum, % households with below poverty level income, Proportion of civilian non-institutionalized population between 18 and 64 who are unemployed, Proportion of household on public assistance % crowded housing, Proportion of households headed by females.	A standardized measure calculated internally at KPNC that capitalizes on readily available U.S. Census Data	-0.32, -0.16 ± 0.84
D4a	Distance to public transit (meters) - measures the minimum walk distance between the population weighted CBG centroid and the nearest transit stop (meters). A distance greater than 3/4 of a mile was assigned a value of -9999.	EPA Smart Location Database	-99999.00, -50163.98 ± 50223.54
publictransit_ind	We created this variable as an interaction term with D4a - to separate people with 0 access to public transit (ie D4a<0). In the Random Forest and LASSO models it was used as an independent variable.	EPA Smart Location Database	0.00, 0.50 ± 0.50
D4c	Aggregate frequency of transit service within 0.25 miles of block group boundary per hour during evening peak period.	EPA Smart Location Database	1.00, 36.92 ± 94.81
D5dri	Regional Centrality Index for Public Transit: CBG D5dr score relative to max CBSA D5dr score. (D5dr score is Employment accessibility by public transit expressed as a ratio of total MSA accessibility)	EPA Smart Location Database	0.00, 0.07 ± 0.14
D5cri	Regional Centrality Index for Automobiles: CBG D5cr score relative to max CBSA D5cr score. (D5cr score is Employment accessibility by automobile expressed as a ratio of total MSA accessibility)	EPA Smart Location Database	0.50, 0.51 ± 0.21
AutoOwn0	Number of households in CBG that own zero automobiles, 2010 decennial Census	EPA Smart Location Database	22.00, 49.51 ± 98.57
LA1and20	A low-income tract with at least 500 people, or 33 percent of the population, living more than 1 mile (urban areas) or more than 20 miles (rural areas) from the nearest supermarket, supercenter, or large grocery store.	United States Department of Agriculture (USDA) Food	0.00, 0.29 ± 0.45

© 2020 Schuler A et al. *JAMA Network Open*.

		Access Research Atlas	
LILATracts_1And10	A low-income tract with at least 500 people, or 33 percent of the population, living more than 1 mile (urban areas) or more than 10 miles (rural areas) from the nearest supermarket, supercenter, or large grocery store.	United States Department of Agriculture (USDA) Food Access Research Atlas	0.00, 0.04 ± 0.21
NatWalkInd	The Walkability Index dataset characterizes every Census 2010 block group in the U.S. based on its relative walkability. Walkability depends upon characteristics of the built environment that influence the likelihood of walking being used as a mode of travel.	EPA Smart Location Mapping	11.17, 11.21 ± 3.93
D1A	Gross residential density (HU/acre) on unprotected land	EPA Smart Location Database	3.96, 5.59 ± 8.50
D1B	Gross population density (people/acre) on unprotected land	EPA Smart Location Database	10.78, 13.80 ± 15.54
D1C	Gross employment density (jobs/acre) on unprotected land	EPA Smart Location Database	1.06, 4.12 ± 20.04
EMPTOT	Total employment in CBSA (CBSA_Emp)	EPA Smart Location Database	194.00, 724.95 ± 2516.62
Ozone	Mean of summer months (May-October) of the daily maximum 8-hour ozone concentration (ppm), averaged over three years (2012 to 2014)	California Communities Environmental Health Screening Tool	0.04, 0.04 ± 0.01
Diesel_PM	Spatial distribution of gridded diesel PM emissions from on-road and non-road sources for a 2012 summer day in July (kg/day).	California Communities Environmental Health Screening Tool	15.98, 20.22 ± 16.72
PM2_5	Annual mean concentration of PM2.5 (average of quarterly means, µg/m3), over three years (2012 to 2014).	California Communities Environmental Health Screening Tool	8.70, 9.13 ± 1.84

© 2020 Schuler A et al. *JAMA Network Open*.

Tox_Releases (RSEIhaz)	Toxicity-weighted concentrations of modeled chemical releases to air from facility emissions and off-site incineration (averaged over 2011 to 2013)	California Communities Environmental Health Screening Tool	219.71, 551.55 ± 2628.18
Traffic	Traffic density – Sum of traffic volumes adjusted by road segment length (vehicle-kilometers per hour) divided by total road length (kilometers) within 150 meters of the census tract boundary (2013)	California Communities Environmental Health Screening Tool	647.81, 826.07 ± 563.37
Haz_Waste	Sum of weighted permitted hazardous waste facilities and hazardous waste generators within each census tract.	California Communities Environmental Health Screening Tool	0.05, 0.47 ± 1.55
Solid_Waste	Sum of weighted solid waste sites and facilities (as of December 2016)	California Communities Environmental Health Screening Tool	0.00, 1.74 ± 4.34
PollutionScore	Pollution Burden scores for each census tract are derived from the average percentiles of the seven Exposures indicators (ozone and PM2.5 concentrations, diesel PM emissions, drinking water contaminants, pesticide use, toxic releases from facilities, and traffic density) and the five Environmental Effects indicators (cleanup sites, impaired water bodies, groundwater threats, hazardous waste facilities and generators, and solid waste sites and facilities). Ranging from 0.1 - 10	California Communities Environmental Health Screening Tool	4.25, 4.43 ± 1.37
DrinkingWater	Drinking water contaminant index for selected contaminants	California Communities Environmental Health Screening Tool	278.76, 347.90 ± 255.36
GroundwaterThreats	Groundwater threats	California Communities Environmental Health Screening Tool	10.00, 22.26 ± 48.40
DM	General areas of drought labeled by intensity. D1 is the least intense level and D4 the most intense. Drought is defined as a moisture deficit bad enough to have social, environmental or economic effects. D0 areas are not in drought but are	California Communities Environmental	4.00, 3.65 ± 0.48

	experiencing abnormally dry conditions that could turn into drought or are recovering from drought but are not yet back to normal.	Health Screening Tool	
2017 Personal Crime Index	Personal Crime Index	Environmental Systems Research Institute (Esri)	70.00, 107.93 ± 117.88
2017 Property Crime Index	Property Crime Index	Environmental Systems Research Institute (Esri)	87.00, 105.66 ± 76.43

**eTABLE 4. URLS OF DATA SOURCES**	
US Department of Agriculture	https://www.ers.usda.gov/data-products/food-access-research-atlas/documentation/#definitions
US Environmental Protection Agency	https://www.epa.gov/sites/production/files/2014-03/documents/sld_userguide.pdf#page=3
California Environmental Protection Agency	https://oehha.ca.gov/media/downloads/calenviroscreen/report/ces3report.pdf
National Oceanic and Atmospheric Administration	https://droughtmonitor.unl.edu/

© 2020 Schuler A et al. *JAMA Network Open*.

eFIGURE. CALIBRATION PLOTS

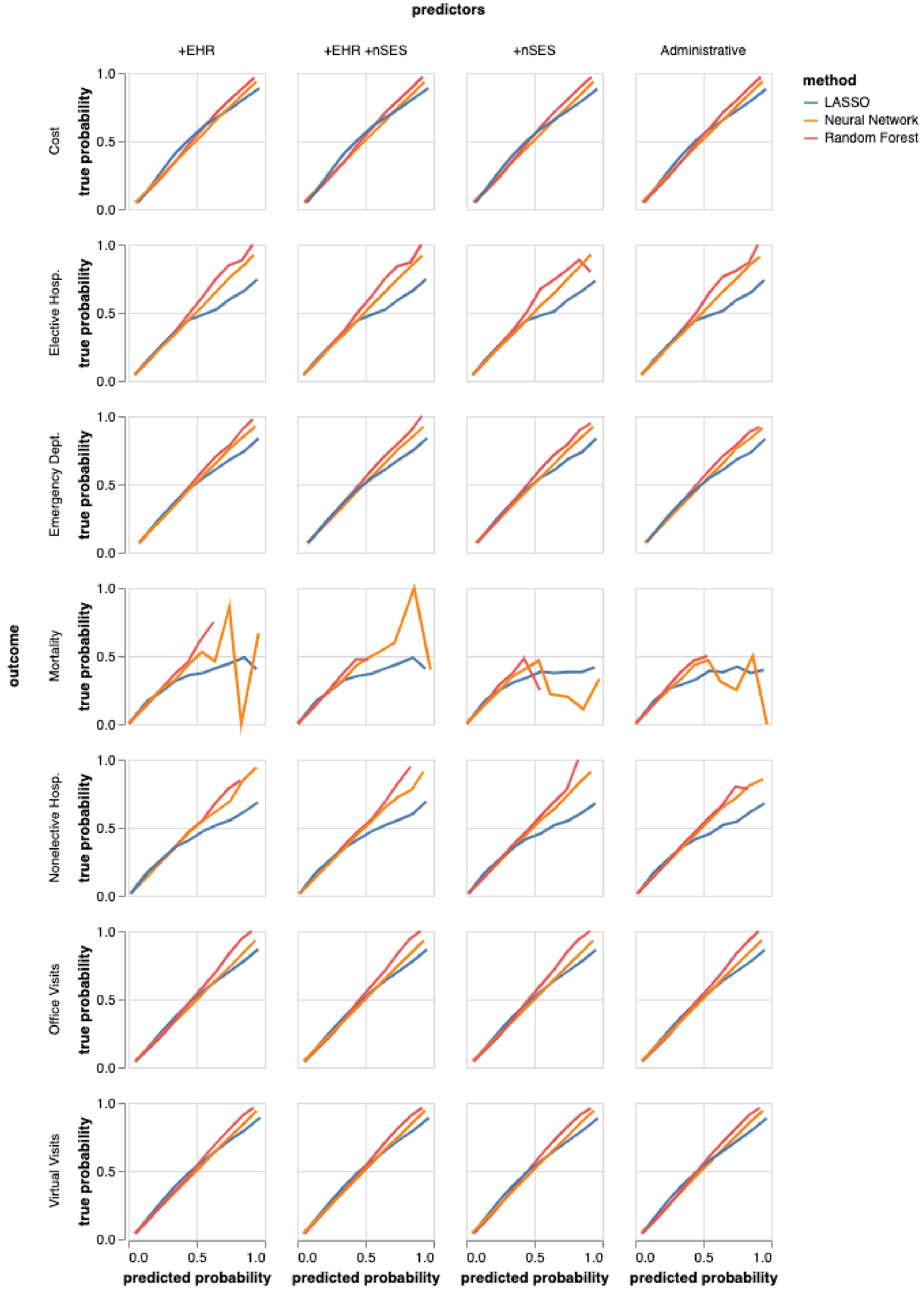

* Calibration in the mortality model deteriorates when the probability > 0.5 because the outcome is quite rare. This is also why the AUPRC statistics for the mortality model are relatively poor.

© 2020 Schuler A et al. *JAMA Network Open*.

eTABLE 5. MODEL OUTCOMES		
**OUTCOME**	**DESCRIPTION**	**80th PERCENTILE**
In-person outpatient visits	All office clinic visits, including addiction & mental health.	≤5 vs ≥6
Virtual visits	Telephone and video visits; scheduled and unscheduled.	≤5 vs ≥6
Emergency department visits	Emergency department visits that did not result in a hospitalization. Includes Emergency Department Treat and Release visits.	0 vs ≥1
Elective hospitalization	Planned hospitalizations (inpatient and observation) that did not begin in the Emergency Department.	0 vs ≥1
Non-elective hospitalization	Unplanned hospitalizations (inpatient and observation) that did begin in the Emergency Department.	0 vs ≥1
Cost	Summary of Ancilliary, Hospital, Scripts, Continuum, Operating Room, Emergency Room and Clinic costs.	<$3,982.21 vs ≥$3982.21
Death	Death in the post period.	-

© 2020 Schuler A et al. *JAMA Network Open*.

eTABLE 6. PERFORMANCE METRICS FOR ALL MODELS						
**OUTCOME**	**METHOD**	**PREDICTORS**	**AUROC**	**AUPRC**	**MCFADDEN R2**	**BRIER SCORE**
Cost	Random Forest	+EHR	0.846	0.649	0.292	0.108
Cost	LASSO	+EHR	0.821	0.590	0.231	0.119
Cost	Neural Network	+EHR	0.847	0.653	0.295	0.108
Cost	Neural Network	+EHR +nSES	0.844	0.646	0.289	0.109
Cost	Random Forest	+EHR +nSES	0.846	0.647	0.291	0.109
Cost	LASSO	+EHR +nSES	0.821	0.590	0.231	0.119
Cost	Random Forest	+nSES	0.846	0.645	0.290	0.109
Cost	Neural Network	+nSES	0.843	0.644	0.287	0.109
Cost	LASSO	+nSES	0.823	0.595	0.235	0.118
Cost	LASSO	Administrative	0.823	0.595	0.235	0.118
Cost	Random Forest	Administrative	0.846	0.647	0.291	0.109
Cost	Neural Network	Administrative	0.847	0.651	0.294	0.108
Mortality	Random Forest	+EHR	0.940	0.227	0.374	0.006
Mortality	LASSO	+EHR	0.933	0.212	0.352	0.006
Mortality	Neural Network	+EHR	0.940	0.219	0.374	0.006
Mortality	Neural Network	+EHR +nSES	0.940	0.222	0.377	0.006
Mortality	Random Forest	+EHR +nSES	0.940	0.224	0.373	0.006
Mortality	LASSO	+EHR +nSES	0.933	0.213	0.354	0.006
Mortality	Random Forest	+nSES	0.938	0.211	0.366	0.006
Mortality	Neural Network	+nSES	0.937	0.204	0.366	0.006
Mortality	LASSO	+nSES	0.932	0.196	0.342	0.006
Mortality	LASSO	Administrative	0.931	0.195	0.341	0.006
Mortality	Random Forest	Administrative	0.939	0.215	0.368	0.006
Mortality	Neural Network	Administrative	0.938	0.211	0.370	0.006
Office Visits	Random Forest	+EHR	0.833	0.579	0.256	0.108
Office Visits	LASSO	+EHR	0.819	0.551	0.228	0.112
Office Visits	Neural Network	+EHR	0.834	0.583	0.259	0.108
Office Visits	Neural Network	+EHR +nSES	0.832	0.580	0.256	0.108
Office Visits	Random Forest	+EHR +nSES	0.833	0.578	0.255	0.109

© 2020 Schuler A et al. *JAMA Network Open*.

eTABLE 6. PERFORMANCE METRICS FOR ALL MODELS						
**OUTCOME**	**METHOD**	**PREDICTORS**	**AUROC**	**AUPRC**	**MCFADDEN R2**	**BRIER SCORE**
Office Visits	LASSO	+EHR +nSES	0.819	0.551	0.228	0.112
Office Visits	Random Forest	+nSES	0.833	0.577	0.255	0.109
Office Visits	Neural Network	+nSES	0.832	0.578	0.255	0.108
Office Visits	LASSO	+nSES	0.818	0.550	0.225	0.113
Office Visits	LASSO	Administrative	0.818	0.550	0.225	0.113
Office Visits	Random Forest	Administrative	0.833	0.578	0.256	0.109
Office Visits	Neural Network	Administrative	0.834	0.582	0.259	0.108
Emergency Dept.	Random Forest	+EHR	0.730	0.396	0.123	0.112
Emergency Dept.	LASSO	+EHR	0.718	0.385	0.110	0.114
Emergency Dept.	Neural Network	+EHR	0.731	0.399	0.125	0.112
Emergency Dept.	Neural Network	+EHR +nSES	0.732	0.401	0.126	0.112
Emergency Dept.	Random Forest	+EHR +nSES	0.731	0.397	0.124	0.112
Emergency Dept.	LASSO	+EHR +nSES	0.722	0.387	0.113	0.114
Emergency Dept.	Random Forest	+nSES	0.730	0.395	0.123	0.112
Emergency Dept.	Neural Network	+nSES	0.730	0.399	0.124	0.112
Emergency Dept.	LASSO	+nSES	0.719	0.385	0.110	0.114
Emergency Dept.	LASSO	Administrative	0.715	0.382	0.107	0.114
Emergency Dept.	Random Forest	Administrative	0.728	0.394	0.122	0.112
Emergency Dept.	Neural Network	Administrative	0.729	0.397	0.124	0.112
Nonelective Hosp.	Random Forest	+EHR	0.854	0.278	0.244	0.027
Nonelective Hosp.	LASSO	+EHR	0.846	0.267	0.229	0.028
Nonelective Hosp.	Neural Network	+EHR	0.856	0.281	0.247	0.027
Nonelective Hosp.	Neural Network	+EHR +nSES	0.855	0.280	0.246	0.027
Nonelective Hosp.	Random Forest	+EHR +nSES	0.853	0.277	0.244	0.027
Nonelective Hosp.	LASSO	+EHR +nSES	0.847	0.267	0.230	0.028
Nonelective Hosp.	Random Forest	+nSES	0.852	0.273	0.241	0.027
Nonelective Hosp.	Neural Network	+nSES	0.854	0.277	0.244	0.027

© 2020 Schuler A et al. *JAMA Network Open*.

eTABLE 6. PERFORMANCE METRICS FOR ALL MODELS						
**OUTCOME**	**METHOD**	**PREDICTORS**	**AUROC**	**AUPRC**	**MCFADDEN R2**	**BRIER SCORE**
Nonelective Hosp.	LASSO	+nSES	0.844	0.263	0.226	0.028
Nonelective Hosp.	LASSO	Administrative	0.844	0.263	0.225	0.028
Nonelective Hosp.	Random Forest	Administrative	0.853	0.274	0.242	0.027
Nonelective Hosp.	Neural Network	Administrative	0.854	0.277	0.245	0.027
Elective Hosp.	Random Forest	+EHR	0.785	0.361	0.173	0.070
Elective Hosp.	LASSO	+EHR	0.765	0.301	0.137	0.074
Elective Hosp.	Neural Network	+EHR	0.786	0.361	0.175	0.070
Elective Hosp.	Neural Network	+EHR +nSES	0.785	0.357	0.172	0.071
Elective Hosp.	Random Forest	+EHR +nSES	0.785	0.360	0.173	0.070
Elective Hosp.	LASSO	+EHR +nSES	0.766	0.301	0.137	0.074
Elective Hosp.	Random Forest	+nSES	0.785	0.356	0.172	0.071
Elective Hosp.	Neural Network	+nSES	0.785	0.352	0.170	0.071
Elective Hosp.	LASSO	+nSES	0.764	0.299	0.135	0.074
Elective Hosp.	LASSO	Administrative	0.763	0.299	0.134	0.074
Elective Hosp.	Random Forest	Administrative	0.785	0.357	0.172	0.071
Elective Hosp.	Neural Network	Administrative	0.786	0.357	0.173	0.071
Virtual Visits	Random Forest	+EHR	0.857	0.630	0.308	0.095
Virtual Visits	LASSO	+EHR	0.847	0.611	0.278	0.098
Virtual Visits	Neural Network	+EHR	0.859	0.633	0.311	0.095
Virtual Visits	Neural Network	+EHR +nSES	0.859	0.633	0.311	0.095
Virtual Visits	Random Forest	+EHR +nSES	0.858	0.631	0.308	0.095
Virtual Visits	LASSO	+EHR +nSES	0.848	0.612	0.280	0.098
Virtual Visits	Random Forest	+nSES	0.856	0.626	0.304	0.096
Virtual Visits	Neural Network	+nSES	0.857	0.628	0.306	0.096
Virtual Visits	LASSO	+nSES	0.846	0.607	0.273	0.099
Virtual Visits	LASSO	Administrative	0.845	0.606	0.271	0.099
Virtual Visits	Random Forest	Administrative	0.856	0.626	0.304	0.096
Virtual Visits	Neural Network	Administrative	0.857	0.628	0.306	0.096

© 2020 Schuler A et al. *JAMA Network Open*.

eAPPENDIX. MODELS

Our neural network is a multi-task feedforward network that simultaneously predicts all outcomes. It was built in the python pytorch framework and trained with the fastai python package. The model was defined by a 9-layer feedforward network with maximum hidden layer of size 1000 and a bottleneck hidden layer of size 10. The training loss across all 7 outcomes was defined as the sum of the outcome-specific cross-entropy losses. The neural net was trained using a batch size of 1000, using the AdamW optimizer with cyclical learning rate and momentum policy and all other hyperparameters left at their defaults. The first layer included a dropout layer at 20%. All layers used BatchNorm and LeakyRelu activation functions, except for the last layer, which used the sigmoid activation function. Our random forest and LASSO models were fit using the h2o python package. Each random forest model used 200 trees and all other parameters were left at their default values. The regularization parameter for the LASSO model was chosen by maximizing AUC in 5-fold cross-validation on the training dataset.

© 2020 Schuler A et al. *JAMA Network Open*.
